# International web-based survey of patients with non-hydrocephalic symptomatic pineal cysts

**DOI:** 10.1007/s00701-024-06403-5

**Published:** 2024-12-28

**Authors:** Jessica Harding, Riccardo Masina, Anna Hill, Ali Ansanipour, Amber Steele, Angelos Kolias, Thomas Santarius

**Affiliations:** 1 Department of Neurosurgery, Addenbrooke’s Hospital, University of Cambridge, Cambridge, UK; 2 Clinical School, University of Cambridge, Cambridge, UK; 3 Heart Centre, Oxford University Hospitals NHS Foundation Trust, Oxford, OX3 9DU UK; 4 Green Templeton College, University of Oxford, Oxford, OX2 6HG UK; 5 Research Strategy & Partnership Hub, Cambridge NIHR Biomedical Research Centre, Cambridge, UK; 6 Pineal Cyst UK, https://www.facebook.com/groups/pinealcystuk/; 7 Department of Clinical Neurosciences, University of Cambridge, Cambridge, UK; 8 Department of Physiology, Development, and Neuroscience, University of Cambridge, Cambridge, UK

**Keywords:** Neurosurgery, Pineal Cyst, Survey, Patient-Reported

## Abstract

**Objectives:**

To report the results of an international patient-reported survey that adds to the growing body of evidence surrounding the role of surgery in the management of a subset of patients with non-hydrocephalic symptomatic pineal cyst.

**Design:**

An international web-based survey of health outcomes in patients with nhSPC.

**Subjects:**

All survey participants who self-reported a diagnosis of symptomatic pineal cyst without hydrocephalus after radiological imaging.

**Methods:**

The survey was developed in collaboration with the patient group Pineal Cyst UK. It was publicised and distributed via several online platforms and social media. Data collected included demographics, cyst size, symptom frequency and severity, number of appointments with healthcare professionals, treatment options trialled, and whether patients underwent surgery.

**Results:**

543 participants (mean age 38.6 years, range 1–83) were included in the analysis, of which 82 (mean age 38.9 years, range 16–72) had undergone cyst resection. After a median period of 18.3 months between date of surgery and date of questionnaire completion, 72 (90%) of the surgical cohort reported overall improvement, and all symptoms improved overall, whereas no symptoms improved overall in the non-surgical cohort. Of the non-surgical cohort (*n* = 461), 269 participants received some form of conservative treatment, of whom 194 (72.1%) did not experience symptom improvement on any treatment offered.

**Conclusions:**

A cohort of patients with nhSPC who participated in this international survey reports substantial and durable improvement in symptom severity and quality of life after pineal cyst resection.

**Supplementary Information:**

The online version contains supplementary material available at 10.1007/s00701-024-06403-5.

## Introduction

Pineal cysts are a common finding on MRI of the brain. A recent study reported a prevalence of 37.5% in healthy individuals with no known neurological or psychiatric illness [15]. The vast majority of the pineal cysts are asymptomatic. Rarely, they present with hydrocephalus and the management of these cysts is well-established [7]. However, the diagnosis, pathophysiology and management of non-hydrocephalic SPCs (nhSPCs) remains controversial [14]. Patients with neurological and/or psychiatric symptoms for whom a cyst without hydrocephalus is discovered on imaging have traditionally been managed conservatively. This is not entirely surprising as the existence of nhSPCs is itself controversial given that there is no generally accepted aetiology to attribute the symptoms to the presence of a pineal cyst. However, there is a growing body of literature reporting symptom improvement following pineal cyst resection, which has led to the relationship between pineal cysts and neuropsychiatric symptoms being re-evaluated. This has also been influenced by an increased understanding of the pathophysiology, with a recent study suggesting that pineal recess crowding can alter blood flow of the internal cerebral veins and cerebrospinal fluid flow and potentially cause brain-wide impairment of glymphatic transport [3].

The first retrospective case series of 18 surgically treated patients with SPCs and without ventriculomegaly or Parinaud’s syndrome was published in 2015 and reported symptom improvement in 94% [10]. Since then, several single-centre retrospective case series have been published and were reviewed in a meta-analysis by Masina et al. (2022), who found an improvement rate of 93% after surgery [12]. However, this evidence comes entirely from single-centre retrospective case series and all outcomes are surgeon-reported. Higher quality of evidence is required to determine the role of surgery in the management of nhSPC.

Patient perspective – a crucial component of effective management of any medical condition – is largely missing from the literature. Here, we report the results from an international, entirely patient-reported, online questionnaire about health outcomes in nhSPC.

## Methods

### Patient and public involvement

The questionnaire was developed in collaboration with Pineal Cyst UK, a patient group represented by Anna Hill, who co-authored this manuscript. The development of the questionnaire was the result of an iterative process, balancing the items of interest to patients and healthcare professionals against the practicalities of survey uptake and delivery of responses. The Project was approved by the Health Research Authority, UK (IRAS 283845, 2020) and HCRW (REC reference 21/NI/0120.

### Data source and study participants

**Fig. 1:**
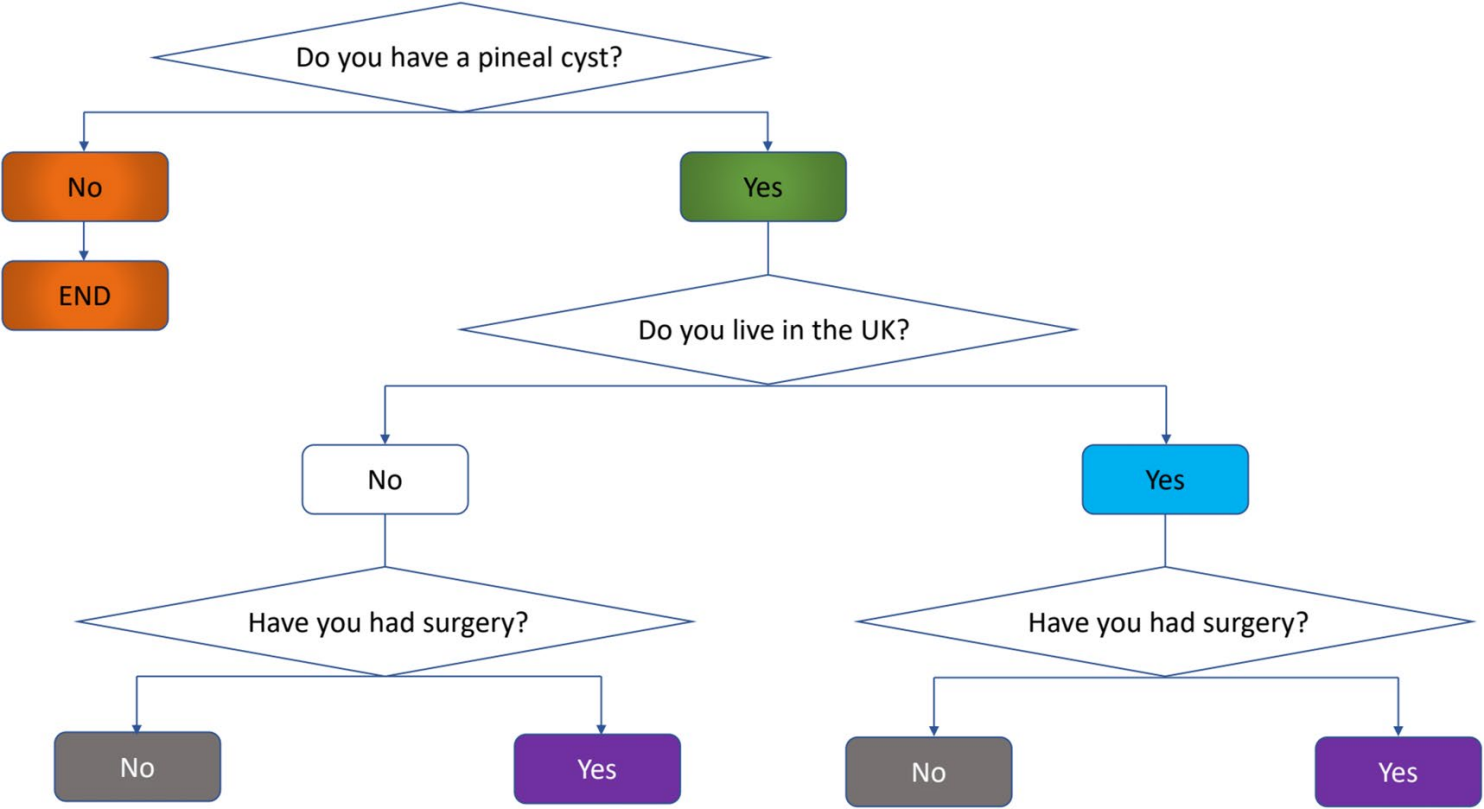
Main pathways of the pineal cyst questionnaire

This retrospective study used data from ‘Symptomatic Pineal Cysts Questionnaire 2020’. It was distributed via several groups on the social media platform Meta (Pineal Cyst UK, Pineal Cyst, Pineal Gland and Tumours, Life after PC Removal, and Pineal Cyst Research) by their members between 1st September 2020 and 13th September 2023 to anyone who was a member of these groups. Included in the study were all patients who indicated that their imaging revealed a pineal cyst and, where available, the size of the cyst was recorded. The broad structure of the questionnaire is represented in Fig. 1. Findings from this study are reported according to STROBE (strengthening the reporting of observational studies in epidemiology) guidelines [4]. The entire questionnaire is available as a Supplement Fig. 1.

### Exposure and predictors

We treated ‘surgery to remove the cyst’ as the exposure, and recorded cyst size (mm, taking the largest diameter), gender, age, and prevalence and severity of symptoms to assess as potential predictor variables.

### Outcome measures

The presence of 13 key symptoms were recorded together with their duration and patient-reported severity (Supplemental Table 1). For the patients who underwent surgical resection, severity measures were assessed ‘prior to’ and ‘after’ surgery, whereas for the non-surgery cohort the same measures were assessed when the symptom ‘first started’ and ‘now’ (i.e. at the time of filling in of the questionnaire). Severity was assessed on a five-point scale: “never had”, “mild”, “moderate”, “severe” and “unbearable”.

Quality of life questions for the surgical cohort asked participants to report the extent of limitations on work and leisure activities, both within the 3 months prior to surgery and at the time of questionnaire. These measures were assessed on a four-point scale: “very much”, “quite a bit”, “a little” and “not at all”. Quality of life after surgery was assessed on a five-point scale: “much better”, “better”, “no change”, “worse” and “much worse”. These measures were only recorded for the surgical cohort. We could not make inter-subgroup comparisons regarding quality-of-life measurements.

### Health-economic impact measurements

We recorded the number of appointments with both general practitioners/family doctors and specialists, separated by speciality. For participants who reported “15 + ” appointments, we treated this as 15 in analysis. We also recorded the number of previous diagnoses and treatments.

### Statistical analyses

**Fig. 2:**
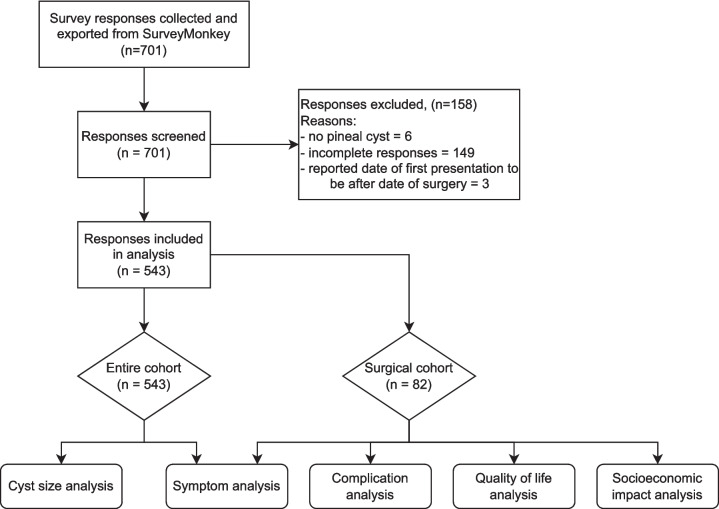
Flowchart of data analysis

Before analysis, the data were filtered by removing participants without pineal cysts, incomplete responses, and participants who reported their date of surgery to be before their first presentation of symptoms. Analyses were conducted according to the pathway represented in Fig. 2, first on the entire cohort, and then on the surgical subgroup. All analyses were performed using R, and we used *p* < 0.05 to determine statistical significance.

Simple descriptive statistics were used to summarise the characteristics of the cohort, and to analyse health-economic impact. Chi-squared tests were used to examine the frequency of symptoms. Given that the data violated the Shapiro–Wilk normality test, Wilcoxon tests were used to investigate differences in cyst size and in self-reported symptom severity between the subgroups. To assess whether cyst size was associated with either the surgical resection of the cyst or any of the 13 key symptoms, linear regression analysis was used.

For the surgical subgroup analysis, paired Wilcoxon tests were used to investigate differences in self-reported symptom severity, and in self-reported quality-of-life measures, before and after surgery. The qualitative responses were converted to numerical scores for analysis. For symptom severity, 1 = “never had” and 5 = “unbearable”, whereas for quality of life, 1 = “much worse” and 5 = “much better”. Associations between complication occurrence and any predictor variables were assessed via logistic regression analysis.

## Results

### Participant characteristics

A total of 543 participants were included in analysis (see Fig. 2). Mean age was 38.6 years (1–83), females predominated 87.7% (476/543), and mean cyst size was 13.7 mm (1.5–35). Of the included 543 participants, 82 underwent surgical resection of the cyst. Of the remaining 461 participants, 269 indicated that they had undergone some form of non-surgical treatment.

For the surgical cohort, mean age was 38.9 years (16–72), females predominated 97.6% (80/82), mean cyst size was 17.0 mm (2–35), and the median time between date of surgery and date of questionnaire completion was 18.3 months (0.5–154.2, reporting median rather than mean because the data violated the Shapiro–Wilk normality test, *p* < 0.001).

**Fig. 3:**
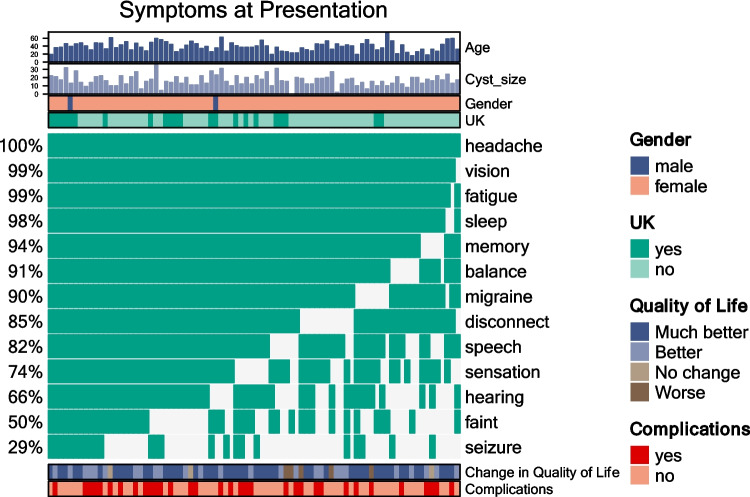
Symptoms at presentation for the surgical cohort

**Fig. 4:**
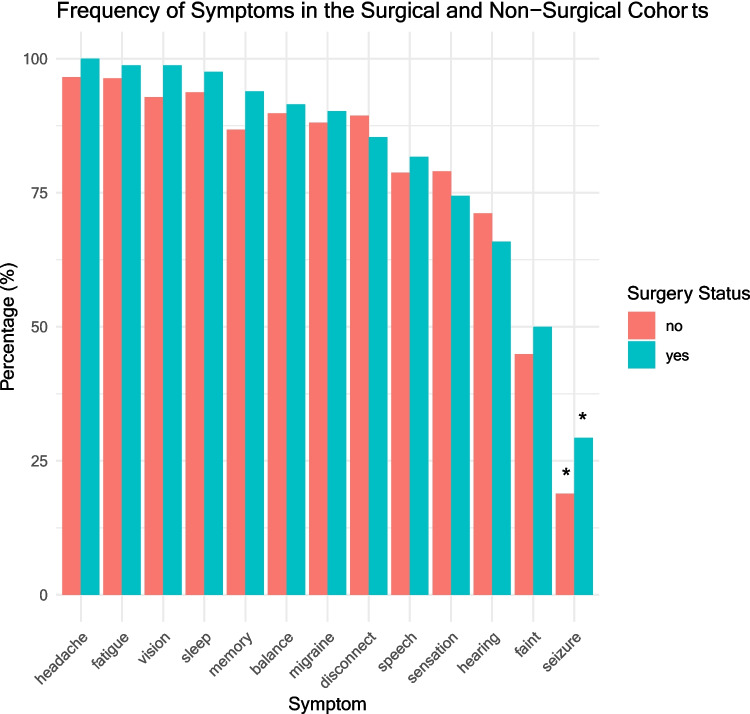
Frequency of symptoms in the surgical and non-surgical cohorts

The characteristics and presenting symptoms for the surgical cohort are listed in Fig. 3. The only symptom for which there was a significant difference in frequency between the surgical and non-surgical cohorts was seizure, which was more common in the surgical cohort (29.3% vs 18.9%, *p* = 0.045, see Fig. 4). There was no significant difference in symptom duration between the surgical and non-surgical cohorts for any of the symptoms measured. For the surgical cohort, mean time between first presentation to the general practitioner or family doctor with symptoms and the date of surgery was 62.7 months.

**Fig. 5:**
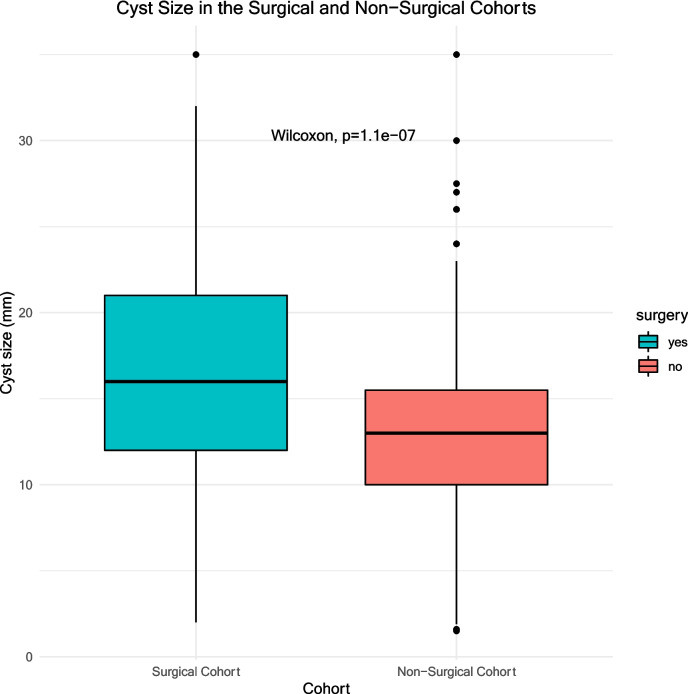
Cyst size measurements in the surgical and non-surgical cohorts

Pineal cyst size was significantly larger in the surgical cohort compared with the non-surgical cohort (Fig. 5, *p* < 0.001). Multivariate linear regression was used to test if any of age, gender or presence of specific symptoms significantly predicted cyst size. Significant associations were found between cyst size and migraine (p = 0.016), and cyst size and seizures (p = 0.007) (see Supplement Table 2).

### Symptom improvement in the surgical cohort

**Fig. 6:**
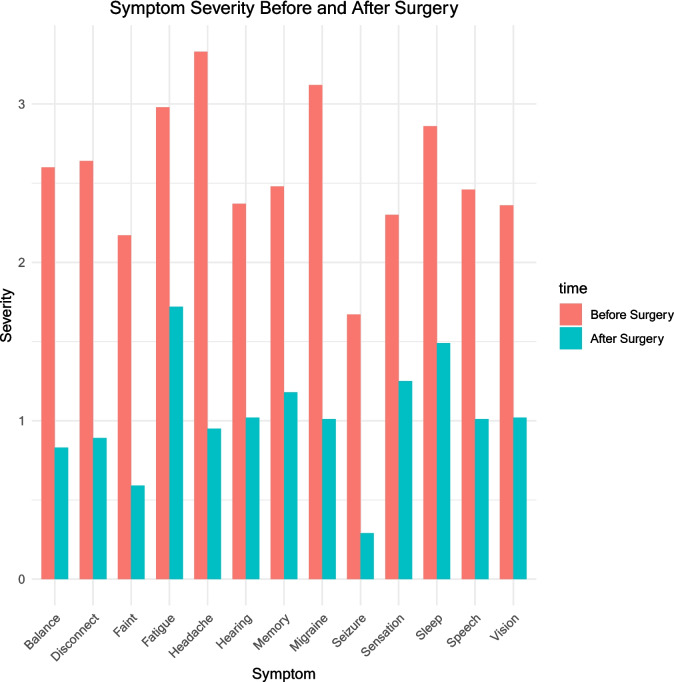
Symptom severity before and after surgery

**Fig. 7:**
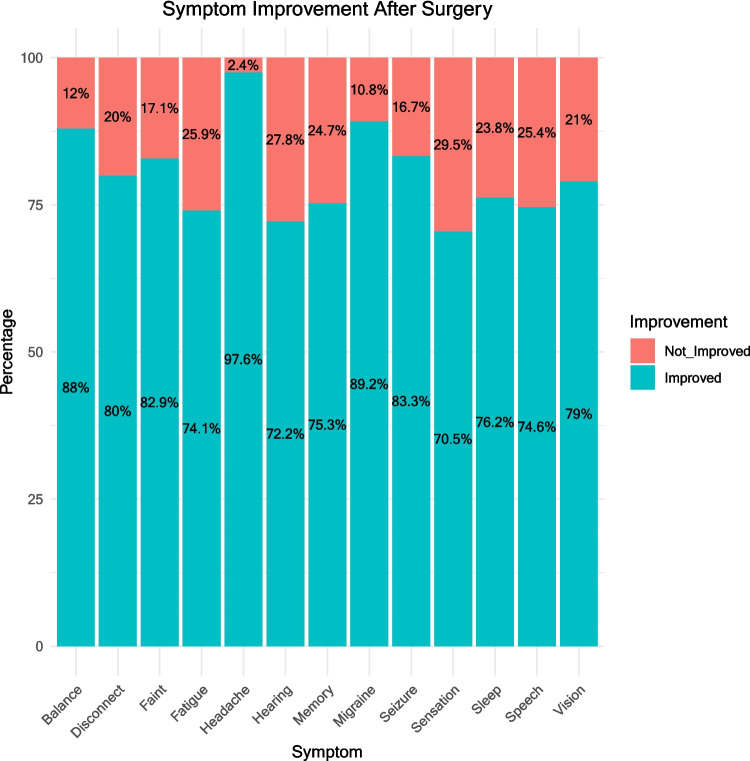
Percentage of the cohort for whom each symptom improved following surgery

For the surgical cohort, on average, there was statistically significant improvement in severity of 13 out of 13 symptoms after surgery (paired Wilcoxon tests, all *p* < 0.001, see Fig. 6). When considering each symptom individually, at least 70% of patients reported improvement in each symptom (Fig. 7) with the most commonly improving symptom being headache in 98% (80/82), and the least common being sensation, improving in 70.5% (43/61).

### Quality of life improvement in the surgical cohort

Surgery resulted in overall improvement (“Much better” or “better” quality of life after surgery) in 90%. There was a significant association (p = 0.026) between quality of life measured after surgery and the difference in extent of limitations on work (although not on leisure) after surgery compared with before. There were significant differences (*p* < 0.001) in the extent of limitations of both work and leisure activities after surgery compared with before. Before surgery, 95% (78/82) were limited in work (“Quite a bit” or “Very much”), compared to 22% (18/82) after surgery.

### Complications of surgery in the surgical cohort

For the surgical cohort, 40% (33/82) of patients replied ‘yes’ in response to ‘Did you have any complications following surgery?’. When then presented with specific categories of complications, 24% selected ‘New neurological disability’ and 11% selected ‘Infection treated with antibiotics’. Eighteen patients (22%) required second surgery: 6 – removal of infected bone flap; 3—insertion of a lumbar drain; 2—external ventricular drain; 1 – eye surgery; 4 patients chose ‘other’. There were no post-operative haematomas requiring evacuation. We were not able to assess whether the complications reported were transient or permanent. There was no association between complication occurrence and self-reported improvement in quality of life. Of those who suffered complications, 76% (25/33) would ‘definitely’ undergo the operation again, despite these, and 82% (27/33) reported overall improvement from surgery (“Much better” or “better” quality of life after surgery).

### Comparison of the surgical cohort with the non-surgical cohort

**Fig. 8:**
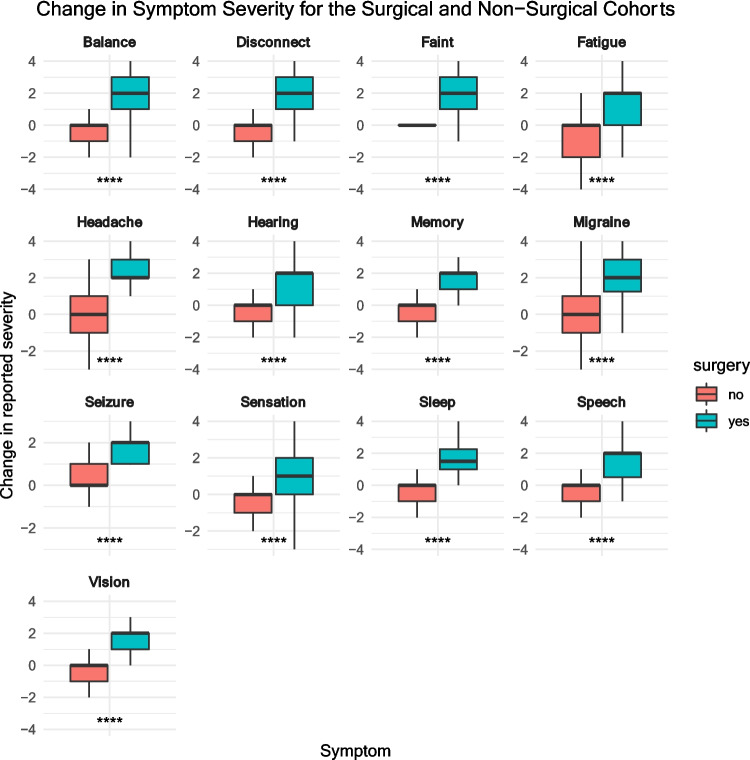
Change in symptom severity in the surgical and non-surgical cohorts. This was calculated by subtracting the numerical ‘severity after surgery’ score from the ‘severity before surgery score’. A positive value indicates improvement in severity, with the magnitude of this change corresponding to the absolute value. For example, in a participant for whom symptom severity was “unbearable” before the operation but “mild” after the operation, change in reported severity would be 5 – 1 = 4

The change in reported symptom severity in the surgical and non-surgical cohorts is represented in Fig. 8. For all symptoms measured, there was a significant (*p* < 0.001) difference in the change in severity between the two cohorts. There was symptom improvement (median change > 0) for every (13 out of 13) symptom in the surgical cohort, compared to 0 out of 13 symptoms in the non-surgical cohort.

For the non-surgical cohort, 72.1% (194/269) did not experience symptom improvement on any treatment offered and 8.9% (24/269) experienced a worsening of symptoms on *all* treatments offered. Average duration of a given treatment was 22 months. Of all participants who received conservative treatment (*n* = 327, including the members of the surgical cohort who were treated with conservative management in the period before they underwent surgery), symptoms did not improve on *any* treatment offered in 70% (229/327), with 30.6% (100/327) experiencing a worsening of symptoms on at least one treatment.

### Health-economic impact

61% (332/543) of participants received at least one alternative diagnosis prior to SPC. The average number of appointments per person with physicians (GP or family doctor) regarding symptoms was 9 (4991/543). Within the surgical cohort, this average was 15 per participant (1209/82). The modal response for the entire cohort was 15 + appointments (*n* = 288, 42% of the cohort). The average number of appointments per person with specialists regarding symptoms was 9 (4731/543), with 17% (94/543) seeing a specialist (from the same specialty) 15 + times. When the surgical cohort was analysed separately, this average increased to 19 per participant (1587/82).

In the surgical cohort, there was a significant difference in the number of participants who had at least 15 appointments with both their GP/family doctor (Before = 47, After = 11, Delta = −36, *p* < 0.001) and with a specialist (Before = 29, After = 8, Delta = −21, *p* < 0.001) before surgery compared with after surgery.

## Discussion

This is the first survey of patient-reported outcomes in patients with nhSPC. Importantly, this strengthens any corroborative results between our study and published, surgeon-reported literature.

### Efficacy of surgery

Overall, 74 survey participants with self-reported nhSPC who underwent surgical resection (90% of the surgical cohort) reported substantial long-term improvement in quality of life at a median time of 18 months after the surgical intervention. The improvement rate of characteristic symptoms of nhSPC, namely headache, visual symptoms and fatigue was 98%, 79% and 74.1%, respectively, which is in line with a recent systematic review and meta-analysis of the retrospective literature [12], as well as with a systematic review focussed on headaches [11]. We found a higher improvement rate for ‘sleep’ symptoms than in the meta-analysis (76.3% compared with 45%). This could be because, where the meta-analysis considered ‘sleep’ symptoms to be ‘sleep disturbance’, we specifically included sleeping ‘too much’ or ‘too little’ as part of any overall ‘issues with sleep’, which could potentially have been underreported in patients asked only about ‘disturbance’. Second, the studies included in the meta-analysis may not have asked patients about sleep at all and/or sleep data may not have been recorded. An alternative explanation is that answers to an online survey via a patient group may be biased by the desire to raise awareness of the severity of the condition.

A major argument against the effectiveness of surgery, especially when subjective outcome measures are assessed, is the placebo effect that is associated with it [6, 8, 16]. Here, participants reportedly remained better 18.3 months after surgery, with 25 participants reporting sustained improvements at least 3 years post-surgery. Whilst this reduces the plausibility of placebo effect being the only explanation for benefit post-surgery, caution is still necessary in interpreting this result because a meta-analysis of temporal changes of the placebo response in surgical trials reported a time-invariant placebo effect for subjective (patient-reported) outcome measures for at least a year [16]. However, this same study found that trials investigating objective outcomes tended to have no clear effect in the placebo arm; further studies, ideally with objective outcome measures, are necessary to explore the extent to which improvement rate is influenced by placebo effect.

### Efficacy of conservative management

Participants reported no benefit from non-surgical management (median change in symptom severity was 0 for 13 out of 13 symptoms, see Fig. 8), and we found that, despite years (on average) of non-surgical management, symptoms did not improve in 72.1% and worsened on at least one treatment in 29% of participants. These results are broadly accordant with the conservatively managed patients in the series by Majovsky et al. (*N* = 110), who found that symptoms were stable in 74% and worsened in 16% [11]. Whilst this could support a degree of inefficacy of conservative management, these results could also be explained by the selection bias inherent to online surveys. For example, it is possible that patients who are better from non-surgical management leave the patient group as they have lost interest. Similarly, only patients who are no better may be motivated enough to fill in this questionnaire, which would bias reporting according to personal desires to raise awareness about the condition.

### Safety of surgery

The complication rate found in this study (40%) was higher than that found in surgeon-reported literature. Masina et al. (2022) reported a complication rate of 17% in their meta-analysis [12]. There are likely several reason for this discrepancy. First, the surveyed surgical cohort may not be representative of the surgical cohort of retrospective studies. Much of this discrepancy could also be related to different perspective of surgical teams and patients [13, 17]. Perspectives of individual surgical teams also differ. Despite efforts to standardise reporting of surgical complication, it is not possible to make any direct comparisons across most surgical procedures [1, 2]. Thirdly, the published case series are affected by publication bias because, in surgery, positive outcomes are heavily overrepresented in the literature [5]. Finally, without providing a clear definition of what constitutes a complication in the survey, it is possible that at least some participants included problems and events that occurred following surgery that would not be considered a complication by the definitions used in surgeon-reported literature. Taken together, these factors can probably account for most of the discrepancy between surgeon- and patient-reported complications. Neither retrospective case series nor surveys are likely to capture a truly representative sample of population who underwent surgery for nhSPC. To definitely address the issue, high-quality prospective evidence is required.

### Health economic implications

These results add patient perspective to the evolving discussion around what role, if any, surgery might have in the management of some patients with nhSPC. This enables some insight into the health-economic implications of nhSPC management strategies. Recent data indicate that the average unit cost to the NHS of outpatient attendances is £235 [9]. For patients seeing a specialist 15 + times, this equates to a cost of at least £3525 (which will be an underestimation, given that, in our analysis, we treated ‘15 + ’ as 15). We found a significant reduction in number of appointments after surgery compared with before. Not only this, but 77% (60/78) of participants who were unable to work before surgery were no longer limited in work post-surgery, which has major quality of life and economic implications. Taken together, these findings indicate that surgery could be cost-effective long-term; healthcare consumption is reduced, and patients are finally able to contribute to the workforce.

### Limitation: selection bias

This study has several limitations. Most importantly, this cohort does not fully represent patients with nhSPC, nor does it represent patients with nhSPC who have undergone surgical resection. For example, it is plausible that this method of data collection resulted in a potential underrepresentation for those patients for whom surgery made no difference, worsened their symptoms, or for whom symptoms are only mild. Any of these populations may be less likely to join patient groups.

### Limitation: study design

By advertising the questionnaire via social media groups, we are unable to gather data on the response rate because it is unknown how many people were presented with the questionnaire. Furthermore, this study is uninformative of serious complications resulting in mortality and severe disability, as well as in assessing the efficacy of the intervention beyond the level of anecdotal evidence.

### Limitation: external validity

Despite the richness of the dataset, the conclusions of this study are limited in their external validity (so in their generalisability outside of the dataset) for several reasons. First, there was heterogeneity of data reporting by participants. For example, whilst we corrected the cyst size measurements for which ‘cm’ units had been included, not all responses included units, so it is possible that some of the smaller measurements were cm measurements rather than mm. This would result in an underestimation of average cyst size, which could have affected analysis of associations.

Second, whilst we can say that, overall, surgery was beneficial for the 82 participants of the surgical cohort who completed the questionnaire, this does not mean that the same would be true for the participants who have not had their pineal cyst resected. Indication for surgery is complex and nuanced, based on clinical and radiological cues as perceived by the surgeon in the context of their experience and individual hypotheses-based understanding of the likely pathogenetic mechanisms involved and thus highly subjective (often including larger cyst size and heavy symptom burden). Clarification of clinical and/or radiological outcome markers and their association with symptoms at presentation is a priority for future research.

Ideally, this future research would be conducted in the form of a randomised controlled trial comparing surgical resection of the pineal cyst with an appropriate placebo operation in a group of symptomatic patients with pineal cysts. However, design and execution of clinical trials on the treatment of pineal cysts would not be straightforward, with potential ethical questions fronting the likely many challenges. A prospective cohort study assessing safety and efficacy of surgery in nhSPC will provide high-quality evidence to shed further light on the condition.

## Conclusion

We have reported the first international web-based patient-reported survey of health outcomes in nhSPC. This adds the patient perspective to the growing body of evidence suggesting a role for surgery in a subset of patients with the condition.

## Supplementary Information

Below is the link to the electronic supplementary material.

Supplementary file1 (PDF 352 KB)

Supplementary file2 (DOCX 180 KB)

## Data Availability

No datasets were generated or analysed during the current study.
