# Supplementary material for: International web-based survey of patients with non-hydrocephalic symptomatic pineal cysts
Source: Acta Neurochir (Wien). 2024 Dec 28;166(1):509. doi: 10.1007/s00701-024-06403-5 (PMC11682015; doi:10.1007/s00701-024-06403-5)
Supplement: Supplementary file 1 — Supplementary file1 (PDF 352 KB) [file 701_2024_6403_MOESM1_ESM.pdf]

Symptomatic Pineal Cysts Questionnaire 2020

Pineal cysts are common, but it seems that only some cause symptoms. As you know, it is difficult to be sure about what symptoms are caused by the cysts and whether removing the cyst is going to improve the symptoms; and if so, with what likelihood.

You have probably seen that the awareness of pineal cysts as a cause of symptoms is variable among healthcare professionals and we would like to learn more about this too.

By filling in this questionnaire you will help us not only understand the symptoms and the role of surgery in the management of symptomatic pineal cysts, but you will also help with the push for improvement of the service for people with symptomatic pineal cysts.

We know that your time is precious but, please, be as accurate as possible in your answers. We tried to keep this survey as brief as possible, yet as useful as possible in helping us achieve our common goals.

Each individual responder’s contribution is equally valuable.

We are grateful for your contribution!

Please note that you can save your progress and return to the survey at any time!

Simply press the **'EXIT'** button at the top right corner to save and exit the survey and when ready to continue, use the survey link on the **same device** to return to your last question.

* Do you have or have you had a pineal cyst?

- ◯ Yes
- ◯ No

Thank you

You have probably come across this in error. There is no need to continue with this questionnaire.

Thank you.

Location

* Do you live in the UK?

◯ Yes

◯ No

About you and your pineal cyst

* Please write the first two letters of your postcode

* Please write the name of the nearest town with a hospital

* What is your gender?

- Male
- Female
- Other (please specify)

* What is your age?

* I am a member of the following Facebook group(s) - please tick all that apply to you:

- Pineal Cyst UK
- Pineal Cyst (private)
- Pineal Gland Cyst and Tumours (public)
- Life After Pineal Cyst
- Other (please specify)

About you and your pineal cyst

* Please write the country in which you reside

[ ]

* Please write the name of the nearest town with a hospital

[ ]

* What is your gender?

- ◯ Male
- ◯ Female
- ◯ Other (please specify)

[ ]

* What is your age?

[ ]

* What is the size of your pineal cyst in millimetres (mm)? (If you have several different measurements/dimensions, please, enter the largest measurement)

[ ]

* Have you had an operation to remove the pineal cyst?

- ◯ Yes
- ◯ No

* I am a member of the following Facebook group(s) - please tick all that apply to you:

- ☐ Pineal Cyst UK
- ☐ Pineal Cyst (private)
- ☐ Pineal Gland Cyst and Tumours (public)
- ☐ Life After Pineal Cyst
- ☐ Other (please specify)

[ ]

About your diagnosis

* When did you first see your general practitioner (GP) with symptoms? (Approximate date is fine, i.e. Month and Year, so please chose a random date within your chosen month)

Date / Time

Date

DD/MM/YYYY

* Did you see an **NHS** neurosurgeon in the UK?

- Yes
- No

* Could you choose the **NHS** neurosurgeon you saw in the UK?

- Yes
- No
- Not applicable

If so, how did you make this decision?

Neurosurgical review

* What advice were you given by this **NHS** neurosurgeon?

- Pineal cysts do not cause symptoms
- Pineal cyst has nothing to do with your symptoms
- Pineal cysts maybe responsible for your symptoms but surgery is too risky
- Pineal cyst is very likely responsible for your symptoms and we are prepared to remove your cyst
- Other (please specify)

* Following this, did you see any other **NHS** neurosurgeons in the UK about your cyst?

- Yes
- No

Other neurosurgical review

* What advice were you given by this **second NHS** neurosurgeon?

- ◯ Pineal cysts do not cause symptoms
- ◯ Pineal cyst has nothing to do with your symptoms
- ◯ Pineal cysts maybe responsible for your symptoms but surgery is too risky
- ◯ Pineal cyst is very likely responsible for your symptoms and we are prepared to remove your cyst
- ◯ Other (please specify)

* Following this, did you see any other **NHS** neurosurgeons in the UK about your cyst?

- ◯ Yes
- ◯ No

Other neurosurgical review

* What advice were you given by this **third NHS** neurosurgeon?

- ◯ Pineal cysts do not cause symptoms
- ◯ Pineal cyst has nothing to do with your symptoms
- ◯ Pineal cysts maybe responsible for your symptoms but surgery is too risky
- ◯ Pineal cyst is very likely responsible for your symptoms and we are prepared to remove your cyst
- ◯ Other (please specify)

* Following this, did you see any other NHS neurosurgeons in the UK about your cyst?

- ◯ Yes
- ◯ No

Other neurosurgical review

Please describe how many **other NHS** neurosurgeons you consulted in the UK and what advice you were given.

Private neurosurgical review

* Did you consult any **private** neurosurgeons in the UK?

- ◯ Yes
- ◯ No

Private neurosurgical review

* What advice were you given by this **private** neurosurgeon?

- ◯ Pineal cysts do not cause symptoms
- ◯ Pineal cyst has nothing to do with your symptoms
- ◯ Pineal cysts maybe responsible for your symptoms but surgery is too risky
- ◯ Pineal cyst is very likely responsible for your symptoms and we are prepared to remove your cyst
- ◯ Other (please specify)

* Following this, did you see any other **private** neurosurgeons in the UK about your cyst?

- ◯ Yes
- ◯ No

Other private neurosurgical review

* What advice were you given by this **second private** neurosurgeon?

- ◯ Pineal cysts do not cause symptoms
- ◯ Pineal cyst has nothing to do with your symptoms
- ◯ Pineal cysts maybe responsible for your symptoms but surgery is too risky
- ◯ Pineal cyst is very likely responsible for your symptoms and we are prepared to remove your cyst
- ◯ Other (please specify)

* Following this, did you see any other **private** neurosurgeons in the UK about your cyst?

- ◯ Yes
- ◯ No

Other private neurosurgical review

Please describe how many other **private** neurosurgeons you consulted in the UK and what advice you were given.

Neurosurgical review outside of the UK

* Did you consult any neurosurgeons **outside of the UK**?

- ◯ Yes
- ◯ No

Neurosurgical review 1 outside of the UK

* Where was this neurosurgeon based?

* Did you send your scans to this neurosurgeon?

- ◯ Yes
- ◯ No

* What was their advice?

- ◯ Pineal cysts do not cause symptoms
- ◯ Pineal cyst has nothing to do with your symptoms
- ◯ Pineal cysts maybe responsible for your symptoms but surgery is too risky
- ◯ Pineal cyst is very likely responsible for your symptoms and we are prepared to remove your cyst

* Did you contact any other neurosurgeons outside of the UK about your cyst?

- ◯ Yes
- ◯ No

Neurosurgical review 2 outside of the UK

* Where was this second neurosurgeon based?

* Did you send your scans to this neurosurgeon?

- Yes
- No

* What was their advice?

- Pineal cysts do not cause symptoms
- Pineal cyst has nothing to do with your symptoms
- Pineal cysts maybe responsible for your symptoms but surgery is too risky
- Pineal cyst is very likely responsible for your symptoms and we are prepared to remove your cyst

* Did you contact any other neurosurgeons outside of the UK about your cyst?

- Yes
- No

Other neurosurgical review outside of the UK

Please describe how many other neurosurgeons you consulted, their locations, and what advice you were given.

* What is/was the size of your pineal cyst in millimetres (mm)? (If you have several different measurements/dimensions, please, enter the largest measurement)

* Have you had an operation to remove the pineal cyst?

- Yes
- No

About your operation

* Please give the date of your operation (Approximate date is fine, i.e. Month and Year, so please chose a random date within your chosen month)

Date / Time

Date

DD/MM/YYYY

* Where was your operation carried out?

* What operation did you have?

- Insertion of a ventriculo-peritoneal shunt only
- Endoscopic third ventriculostomy only
- Removal/resection of the cyst through an incision on the back of my head (endoscopic)
- Removal/resection of the cyst through an incision on the back of my head (microscopic)
- Removal/resection of the cyst through an incision on the back of my head (unsure of the approach)
- Other (please specify)

What was the total cost of your operation, including travel and accommodation expenses? Please round up to the nearest £1,000 (Optional)

How did you pay for your surgery? (Optional)

- Used own money/savings
- Fundraising e.g. GoFundMe
- Bank loan/other kinds of loan
- Other (please specify)

* After your operation, did you require a second procedure?

- Yes
- No

If yes, please select all that applies.

- [ ] Insertion of a ventriculo-peritoneal shunt
- [ ] Endoscopic third ventriculostomy
- [ ] Insertion of a temporary drain
- [ ] Operation to remove infected bone flap
- [ ] Other (please specify)

* Did you have any complications following surgery?

- ( ) Yes
- ( ) No

If yes, please select all that applies.

- [ ] New neurological disability
- [ ] Infection (treated with antibiotics only)
- [ ] Infection (requiring another operation to remove infected bone flap)
- [ ] Bleeding that required a separate operation
- [ ] Hydrocephalus (requiring temporary external drain)
- [ ] Hydrocephalus (requiring insertion of a ventriculoperitoneal shunt)
- [ ] Other (please specify)

* Do you currently suffer from any wound pain?

- ( ) No
- ( ) Yes, but don’t require regular painkillers
- ( ) Yes, but it is manageable with simple painkillers (paracetamol, non-steroidal painkillers like ibuprofen etc)
- ( ) Yes, but it is manageable with the help of pain special painkillers, such as gabapentin, pregabalin, amitriptyline etc
- ( ) The pain is not controlled and is a major issue

* Did you require to see a pain specialist after the operation?

- ( ) No
- ( ) I had to see a pain specialist once
- ( ) I had to see a pain specialist 2 times
- ( ) I had to see a pain specialist 3 times
- ( ) I had to see a pain specialist more than 3 times

* How does your quality of life after surgery compare with before surgery?

Much worse	Worse	No change	Better	Much better
( )	( )	( )	( )	( )

* Were you limited in doing either your work or other daily activities for the last 3 months BEFORE your operation?

- Not at all
- A little
- Quite a bit
- Very much

* Were you limited in pursuing your hobbies or other leisure time activities for the last 3 months BEFORE your operation?

- Not at all
- A little
- Quite a bit
- Very much

* Are you CURRENTLY limited in doing either your work or other daily activities?

- Not at all
- A little
- Quite a bit
- Very much

* Are you CURRENTLY limited in pursuing your hobbies or other leisure time activities?

- Not at all
- A little
- Quite a bit
- Very much

* Overall, would you have chosen to undergo the operation again knowing what you know today?

Definitely yes	Very likely	Possibly	Very unlikely	Definitely no
○	○	○	○	○

About your operation

* When did you first present to your family doctor with symptoms? (Approximate date is fine, i.e. Month and Year, so please chose a random date within your chosen month)

Date / Time

Date

DD/MM/YYYY

* Please give the date of your operation (Approximate date is fine, i.e. Month and Year, so please chose a random date within your chosen month)

Date / Time

Date

DD/MM/YYYY

* Where was your operation carried out?

* What operation did you have?

- ○ Insertion of a ventriculo-peritoneal shunt only
- ○ Endoscopic third ventriculostomy only
- ○ Removal/resection of the cyst through an incision on the back of my head (endoscopic)
- ○ Removal/resection of the cyst through an incision on the back of my head (microscopic)
- ○ Removal/resection of the cyst through an incision on the back of my head (unsure of the approach)
- ○ Other (please specify)

What was the total cost of your operation, including travel and accommodation expenses? Please round up to the nearest £1,000 (Optional)

How did you pay for your surgery? (Optional)

- ☐ Used own money/savings
- ☐ Fundraising e.g. GoFundMe
- ☐ Bank loan/other kinds of loan
- ☐ Other (please specify)

* After your operation, did you require a second procedure?

- Yes
- No

If yes, please select all that applies.

- Insertion of a ventriculo-peritoneal shunt
- Endoscopic third ventriculostomy
- Insertion of a temporary drain
- Operation to remove infected bone flap
- Other (please specify)

* Did you have any complications following surgery?

- Yes
- No

If yes, please select all that applies.

- New neurological disability
- Infection (treated with antibiotics only)
- Infection (requiring another operation to remove infected bone flap)
- Bleeding that required a separate operation
- Hydrocephalus (requiring temporary external drain)
- Hydrocephalus (requiring insertion of a ventriculoperitoneal shunt)
- Other (please specify)

* Do you currently suffer from any wound pain?

- No
- Yes, but don’t require regular painkillers
- Yes, but it is manageable with simple painkillers (paracetamol, non-steroidal painkillers like ibuprofen etc)
- Yes, but it is manageable with the help of pain special painkillers, such as gabapentin, pregabalin, amitriptyline etc
- The pain is not controlled and is a major issue

* Did you require to see a pain specialist after the operation?

- No
- I had to see a pain specialist once
- I had to see a pain specialist 2 times
- I had to see a pain specialist 3 times
- I had to see a pain specialist more than 3 times

* How does your quality of life after surgery compare with before surgery?

Much worse	Worse	No change	Better	Much better
◯	◯	◯	◯	◯

* Were you limited in doing either your work or other daily activities for the last 3 months BEFORE your operation?

◯ Not at all

◯ A little

◯ Quite a bit

◯ Very much

* Were you limited in pursuing your hobbies or other leisure time activities for the last 3 months BEFORE your operation?

◯ Not at all

◯ A little

◯ Quite a bit

◯ Very much

* Are you CURRENTLY limited in doing either your work or other daily activities?

◯ Not at all

◯ A little

◯ Quite a bit

◯ Very much

* Are you CURRENTLY limited in pursuing your hobbies or other leisure time activities?

◯ Not at all

◯ A little

◯ Quite a bit

◯ Very much

* Overall, would you have chosen to undergo the operation again knowing what you know today?

Definitely yes	Very likely	Possibly	Very unlikely	Definitely no
◯	◯	◯	◯	◯

About your symptoms

We would like to know more about your specific symptoms and if they have changed after the operation.

For each symptom mentioned, please answer the questions using the scale provided.

Headache or "pressure in the head"-like sensation

* Have you experienced any **headache or "pressure in the head"-like sensation**?

- ◯ Yes
- ◯ No (Below please chose for “Duration" -> "Not applicable" and for "How severe...” -> “Never had”)

* Duration

[ ]

* How severe was this symptom prior to the operation?

Never had	Mild	Moderate	Severe	Unbearable
◯	◯	◯	◯	◯

* How severe is this symptom after the operation?

Never had	Mild	Moderate	Severe	Unbearable
◯	◯	◯	◯	◯

Migraine-like headaches

* Have you experienced any **migraine-like headaches**?

- ◯ Yes
- ◯ No (Below please chose for “Duration" -> "Not applicable" and for "How severe…” -> “Never had”)

If yes, do you have auras before your headaches?

- ◯ Never
- ◯ Sometimes
- ◯ Always

* Duration

[ ]

* How severe was this symptom prior to the operation?

Never had	Mild	Moderate	Severe	Unbearable
◯	◯	◯	◯	◯

* How severe is this symptom after the operation?

Never had	Mild	Moderate	Severe	Unbearable
◯	◯	◯	◯	◯

Visual symptoms

* Have you experienced any **visual symptoms**?

- ◯ Yes
- ◯ No (Below please chose for “Duration" -> "Not applicable" and for "How severe...” -> “Never had”)

If yes, tick any that apply to you

- ☐ blurred vision
- ☐ double vision
- ☐ light sensitivity
- ☐ Other (please specify)

____

* Duration

____

* How severe was this symptom prior to the operation?

Never had	Mild	Moderate	Severe	Unbearable
◯	◯	◯	◯	◯

* How severe is this symptom after the operation?

Never had	Mild	Moderate	Severe	Unbearable
◯	◯	◯	◯	◯

Hearing problems

* Have you experienced any **hearing problems**?

- ○ Yes
- ○ No (Below please chose for “Duration" -> "Not applicable" and for "How severe...” -> “Never had”)

If yes, tick any that apply to you

- ☐ reduced hearing
- ☐ hearing noises
- ☐ hearing voices
- ☐ Other (please specify)

* Duration

* How severe was this symptom prior to the operation?

Never had	Mild	Moderate	Severe	Unbearable
○	○	○	○	○

* How severe is this symptom after the operation?

Never had	Mild	Moderate	Severe	Unbearable
○	○	○	○	○

Balance problems

* Have you experienced any **balance problems**?

- ◯ Yes
- ◯ No (Below please chose for “Duration" -> "Not applicable" and for "How severe…” -> “Never had”)

If yes, tick any that apply to you

- ☐ Poor balance
- ☐ I have a sensation of movement - as if I was on a rotating chair/the room is spinning
- ☐ I feel like have no control of my legs
- ☐ I feel light-headed
- ☐ Other (please specify)

* Duration

* How severe was this symptom prior to the operation?

Never had	Mild	Moderate	Severe	Unbearable
◯	◯	◯	◯	◯

* How severe is this symptom after the operation?

Never had	Mild	Moderate	Severe	Unbearable
◯	◯	◯	◯	◯

Speech problems

* Have you experienced any **speech problems**?

- Yes
- No (Below please chose for “Duration" -> "Not applicable" and for "How severe...” -> “Never had”)

If yes, tick any that apply to you

- Difficulty with choosing of the right words
- No problem with thinking of the words but problem saying it – slurred speech
- Other (please specify)

* Duration

* How severe was this symptom prior to the operation?

Never had	Mild	Moderate	Severe	Unbearable
○	○	○	○	○

* How severe is this symptom after the operation?

Never had	Mild	Moderate	Severe	Unbearable
○	○	○	○	○

Cognitive/memory problems

* Have you experienced any **cognitive/memory problems**?

- ◯ Yes
- ◯ No (Below please chose for “Duration" -> "Not applicable" and for "How severe…” -> “Never had”)

If yes, tick any that apply to you

- ☐ Difficulty remembering words
- ☐ Difficulty remembering events
- ☐ Difficulty remembering numbers
- ☐ Other (please specify)

* Duration

* How severe was this symptom prior to the operation?

Never had	Mild	Moderate	Severe	Unbearable
◯	◯	◯	◯	◯

* How severe is this symptom after the operation?

Never had	Mild	Moderate	Severe	Unbearable
◯	◯	◯	◯	◯

Disconnection

* Have you experienced any "**feeling of disconnection**"?

- ◯ Yes
- ◯ No (Below please chose for “Duration" -> "Not applicable" and for "How severe...” -> “Never had”)

If yes, tick any that apply to you

- ☐ Feeling of disconnection from the surrounding
- ☐ Woolly brain
- ☐ Foggy brain
- ☐ Other (please specify)

* Duration

* How severe was this symptom prior to the operation?

Never had	Mild	Moderate	Severe	Unbearable
◯	◯	◯	◯	◯

* How severe is this symptom after the operation?

Never had	Mild	Moderate	Severe	Unbearable
◯	◯	◯	◯	◯

Sleep problems

* Have you experienced any **issues with sleep**?

○ Yes

○ No (Below please chose for “Duration" -> "Not applicable" and for "How severe…” -> “Never had”)

If yes, tick any that apply to you

☐ I sleep too much

☐ I sleep too little

☐ I wake frequently during sleep

☐ Other (please specify)

* Duration

* How severe was this symptom prior to the operation?

Never had	Mild	Moderate	Severe	Unbearable
○	○	○	○	○

* How severe is this symptom after the operation?

Never had	Mild	Moderate	Severe	Unbearable
○	○	○	○	○

Fainting

* Have you experienced any **episode of fainting**?

- ◯ Yes
- ◯ No (Below please chose for “Duration" -> "Not applicable" and for "How severe…” -> “Never had”)

If yes, tick any that apply to you

- ☐ Sudden loss of consciousness
- ☐ Feeling of light-headedness and collapse but no loss of consciousness
- ☐ Other (please specify)

* Duration

* How severe was this symptom prior to the operation?

Never had	Mild	Moderate	Severe	Unbearable
◯	◯	◯	◯	◯

* How severe is this symptom after the operation?

Never had	Mild	Moderate	Severe	Unbearable
◯	◯	◯	◯	◯

Seizure/epileptic attack

* Have you experienced any **seizure/epileptic attacks**?

◯ Yes

◯ No (Below please chose for “Duration" -> "Not applicable" and for "How severe...” -> “Never had”)

If yes, tick any that apply to you

☐ I have been having epilepsy because of some other known cause

☐ I have had LESS than 2 epileptic seizures of unknown cause

☐ I have had MORE than 2 epileptic seizures of unknown cause

☐ Other (please specify)

* Duration

* How severe was this symptom prior to the operation?

Never had	Mild	Moderate	Severe	Unbearable
◯	◯	◯	◯	◯

* How severe is this symptom after the operation?

Never had	Mild	Moderate	Severe	Unbearable
◯	◯	◯	◯	◯

Energy levels/fatigue

* Have you experienced any **low energy levels/fatigue**?

- ◯ Yes
- ◯ No (Below please chose for “Duration" -> "Not applicable" and for "How severe...” -> “Never had”)

If yes, tick any that apply to you

- ☐ I have no energy in the morning
- ☐ I have energy in the morning
- ☐ I have no energy in the evening
- ☐ I have energy in the evening
- ☐ I am constantly fatigued
- ☐ My energy levels are not affected
- ☐ Other (please specify)

* Duration

* How severe was this symptom prior to the operation?

Never had	Mild	Moderate	Severe	Unbearable
◯	◯	◯	◯	◯

* How severe is this symptom after the operation?

Never had	Mild	Moderate	Severe	Unbearable
◯	◯	◯	◯	◯

Sensation problems

* Have you experienced any **sensation problems**?

◯ Yes

◯ No (Below please chose for “Duration" -> "Not applicable" and for "How severe...” -> “Never had”)

If yes, tick any that apply to you

☐ Issues with touch/dexterity
☐ Issues sensing hot
☐ Issues sensing cold
☐ Other (please specify)
☐ Tingling sensations
☐ Difficulty regulating temperature

* Duration

* How severe was this symptom prior to the operation?

Never had	Mild	Moderate	Severe	Unbearable
◯	◯	◯	◯	◯

* How severe is this symptom after the operation?

Never had	Mild	Moderate	Severe	Unbearable
◯	◯	◯	◯	◯

Other symptoms?

Please use this box to add any other symptoms that you have experienced.

Clinician review of your symptoms

How many times have you seen your GP/family doctor for ANY of your symptoms?

	Before the operation	After the operation
Number of visits		

How many times have you seen a specialist for ANY of your symptoms?

	Before the operation	After the operation
Neurologist (NHS)		
Neurologist (Private)		
ENT surgeon (NHS)		
ENT surgeon (Private)		
Ophthalmologist (NHS)		
Ophthalmologist (Private)		
Neurosurgeon (NHS)		
Neurosurgeon (Private)		

About your symptoms

We would like to know more about your specific symptoms and if they have changed over time.

For each symptom mentioned, please answer the questions using the scale provided.

Headache or "pressure in the head"-like sensation

* Have you experienced any **headache or "pressure in the head"-like sensation**?

- Yes
- No (Below please chose for “Duration" -> "Not applicable" and for "How severe...” -> “Never had”)

* Duration

* How severe was this symptom when it first started?

Never had	Mild	Moderate	Severe	Unbearable
○	○	○	○	○

* Compared to when this symptom first started, the symptom is now:

Never had	Mild	Moderate	Severe	Unbearable
○	○	○	○	○

Migraine-like headaches

* Have you experienced any **migraine-like headaches**?

- Yes
- No (Below please chose for “Duration" -> "Not applicable" and for "How severe...” -> “Never had”)

If yes, do you have auras before your headaches?

- Never
- Sometimes
- Always

* Duration

* How severe was this symptom when it first started?

Never had	Mild	Moderate	Severe	Unbearable
○	○	○	○	○

* Compared to when this symptom first started, the symptom is now:

Never had	Mild	Moderate	Severe	Unbearable
○	○	○	○	○

Visual symptoms

* Have you experienced any **visual symptoms**?

◯ Yes

◯ No (Below please chose for “Duration" -> "Not applicable" and for "How severe...” -> “Never had”)

If yes, tick any that apply to you

☐ blurred vision

☐ double vision

☐ light sensitivity

☐ Other (please specify)

* Duration

* How severe was this symptom when it first started?

Never had	Mild	Moderate	Severe	Unbearable
◯	◯	◯	◯	◯

* Compared to when this symptom first started, the symptom is now:

Never had	Mild	Moderate	Severe	Unbearable
◯	◯	◯	◯	◯

Hearing problems

* Have you experienced any **hearing problems**?

- ◯ Yes
- ◯ No (Below please chose for “Duration" -> "Not applicable" and for "How severe...” -> “Never had”)

If yes, tick any that apply to you

- ☐ reduced hearing
- ☐ hearing noises
- ☐ hearing voices
- ☐ Other (please specify)

* Duration

* How severe was this symptom when it first started?

Never had	Mild	Moderate	Severe	Unbearable
◯	◯	◯	◯	◯

* Compared to when this symptom first started, the symptom is now:

Never had	Mild	Moderate	Severe	Unbearable
◯	◯	◯	◯	◯

Balance problems

* Have you experienced any **balance problems**?

- ◯ Yes
- ◯ No (Below please chose for “Duration" -> "Not applicable" and for "How severe...” -> “Never had”)

If yes, tick any that apply to you

- ☐ Poor balance
- ☐ I have a sensation of movement - as if I was on a rotating chair/the room is spinning
- ☐ I feel like have no control of my legs
- ☐ I feel light-headed
- ☐ Other (please specify)

* Duration

* How severe was this symptom when it first started?

Never had	Mild	Moderate	Severe	Unbearable
◯	◯	◯	◯	◯

* Compared to when this symptom first started, the symptom is now:

Never had	Mild	Moderate	Severe	Unbearable
◯	◯	◯	◯	◯

Speech problems

* Have you experienced any **speech problems**?

- ◯ Yes
- ◯ No (Below please chose for “Duration" -> "Not applicable" and for "How severe...” -> “Never had”)

If yes, tick any that apply to you

- ☐ Difficulty with choosing of the right words
- ☐ No problem with thinking of the words but problem saying it – slurred speech
- ☐ Other (please specify)

* Duration

* How severe was this symptom when it first started?

Never had	Mild	Moderate	Severe	Unbearable
◯	◯	◯	◯	◯

* Compared to when this symptom first started, the symptom is now:

Never had	Mild	Moderate	Severe	Unbearable
◯	◯	◯	◯	◯

Cognitive/memory problems

* Have you experienced any **cognitive/memory problems**?

- ◯ Yes
- ◯ No (Below please chose for “Duration" -> "Not applicable" and for "How severe...” -> “Never had”)

If yes, tick any that apply to you

- ☐ Difficulty remembering words
- ☐ Difficulty remembering events
- ☐ Difficulty remembering numbers
- ☐ Other (please specify)

* Duration

* How severe was this symptom when it first started?

Never had	Mild	Moderate	Severe	Unbearable
◯	◯	◯	◯	◯

* Compared to when this symptom first started, the symptom is now:

Never had	Mild	Moderate	Severe	Unbearable
◯	◯	◯	◯	◯

Disconnection

* Have you experienced any "**feeling of disconnection**"?

- ○ Yes
- ○ No (Below please chose for “Duration" -> "Not applicable" and for "How severe...” -> “Never had”)

If yes, tick any that apply to you

- ☐ Feeling of disconnection from the surrounding
- ☐ Woolly brain
- ☐ Foggy brain
- ☐ Other (please specify)

* Duration

* How severe was this symptom when it first started?

Never had	Mild	Moderate	Severe	Unbearable
○	○	○	○	○

* Compared to when this symptom first started, the symptom is now:

Never had	Mild	Moderate	Severe	Unbearable
○	○	○	○	○

Sleep problems

* Have you experienced any **sleep problems**?

- ○ Yes
- ○ No (Below please chose for “Duration" -> "Not applicable" and for "How severe...” -> “Never had”)

If yes, tick any that apply to you

- ☐ I sleep too much
- ☐ I sleep too little
- ☐ I wake frequently during sleep
- ☐ Other (please specify)

* Duration

* How severe was this symptom when it first started?

Never had	Mild	Moderate	Severe	Unbearable
○	○	○	○	○

* Compared to when this symptom first started, the symptom is now:

Never had	Mild	Moderate	Severe	Unbearable
○	○	○	○	○

Fainting

* Have you experienced any **episode of fainting**?

- ◯ Yes
- ◯ No (Below please chose for “Duration" -> "Not applicable" and for "How severe...” -> “Never had”)

If yes, tick any that apply to you

- ☐ Sudden loss of consciousness
- ☐ Feeling of light-headedness and collapse but no loss of consciousness
- ☐ Other (please specify)

* Duration

* How severe was this symptom when it first started?

Never had	Mild	Moderate	Severe	Unbearable
◯	◯	◯	◯	◯

* Compared to when this symptom first started, the symptom is now:

Never had	Mild	Moderate	Severe	Unbearable
◯	◯	◯	◯	◯

Seizure/epileptic attack

* Have you experienced any **seizure/epileptic attacks**?

◯ Yes

◯ No (Below please chose for “Duration" -> "Not applicable" and for "How severe…” -> “Never had”)

If yes, tick any that apply to you

☐ I have been having epilepsy because of some other known cause

☐ I have had LESS than 2 epileptic seizures of unknown cause

☐ I have had MORE than 2 epileptic seizures of unknown cause

☐ Other (please specify)

* Duration

* How severe was this symptom when it first started?

Never had	Mild	Moderate	Severe	Unbearable
◯	◯	◯	◯	◯

* Compared to when this symptom first started, the symptom is now:

Never had	Mild	Moderate	Severe	Unbearable
◯	◯	◯	◯	◯

Energy levels/fatigue

* Have you experienced any **low energy levels/fatigue**?

- ◯ Yes
- ◯ No (Below please chose for “Duration" -> "Not applicable" and for "How severe...” -> “Never had”)

If yes, tick any that apply to you

- ☐ I have no energy in the morning
- ☐ I have energy in the morning
- ☐ I have no energy in the evening
- ☐ I have energy in the evening
- ☐ I am constantly fatigued
- ☐ My energy levels are not affected
- ☐ Other (please specify)

* Duration

* How severe was this symptom when it first started?

Never had	Mild	Moderate	Severe	Unbearable
◯	◯	◯	◯	◯

* Compared to when this symptom first started, the symptom is now:

Never had	Mild	Moderate	Severe	Unbearable
◯	◯	◯	◯	◯

Sensation problems

* Have you experienced any **sensation problems**?

- Yes
- No (Below please chose for “Duration" -> "Not applicable" and for "How severe...” -> “Never had”)

If yes, tick any that apply to you

- Issues with touch/dexterity
- Issues sensing hot
- Issues sensing cold
- Other (please specify)
- Tingling sensations
- Difficulty regulating temperature

* Duration

* How severe was this symptom when it first started?

Never had	Mild	Moderate	Severe	Unbearable
○	○	○	○	○

* Compared to when this symptom first started, the symptom is now:

Never had	Mild	Moderate	Severe	Unbearable
○	○	○	○	○

Other symptoms?

Please use this box to add any other symptoms that you have experienced.

Clinician review of your symptoms

How many times have you seen your GP/family doctor for ANY of your symptoms?

How many times have you seen a specialist for ANY of your symptoms?

	Approximate number of times you have seen this specialist
Neurologist (NHS)	
Neurologist (Private)	
ENT surgeon (NHS)	
ENT surgeon (Private)	
Ophthalmologist (NHS)	
Ophthalmologist (Private)	
Neurosurgeon (NHS)	
Neurosurgeon (Private)	

Diagnosis and treatments

This section will relate to the diagnoses that you were given by your GP/family doctor or your specialist. We also want to learn about the treatment(s) you were given, how long you received these treatments for and how effective they were.

Your diagnoses

Diagnosis 1 Please indicate what diagnosis you were given first.

When did you first receive this diagnosis?

Date / Time

Date

DD/MM/YYYY

Diagnosis 2 Please indicate what diagnosis you were given first.

When did you first receive this diagnosis?

Date / Time

Date

DD/MM/YYYY

Diagnosis 3 Please indicate what diagnosis you were given first.

When did you first receive this diagnosis?

Date / Time

Date

DD/MM/YYYY

Please use this box, as you wish, to describe any other diagnoses you have been given.

Treatments

Treatment 1 Please indicate what treatment you first received. (If applicable)

How long did/have you receive(d) this treatment?

As a result of this treatment my main symptoms are:

Much worse	Worse	No change	Better	Much better
○	○	○	○	○

As a result of this treatment my quality of life is:

Much worse	Worse	No change	Better	Much better
○	○	○	○	○

Treatment 2 Please indicate what treatment you first received. (If applicable)

How long did/have you receive(d) this treatment?

As a result of this treatment my main symptoms are:

Much worse	Worse	No change	Better	Much better
○	○	○	○	○

As a result of this treatment my quality of life is:

Much worse	Worse	No change	Better	Much better
○	○	○	○	○

Treatment 3 Please indicate what treatment you first received. (If applicable)

How long did/have you receive(d) this treatment?

As a result of this treatment my main symptoms are:

Much worse	Worse	No change	Better	Much better
○	○	○	○	○

As a result of this treatment my quality of life is:

Much worse	Worse	No change	Better	Much better
○	○	○	○	○

Please use this box, as you wish, to describe any other treatments you have received.

Thank you for filling in the survey so far. We are designing a study in the UK to better understand and improve the management of patients with symptomatic pineal cysts and we are interested to hear your views.

Do you currently reside in the UK?

- ○ Yes
- ○ No

Your views on the study we are designing

We are preparing a study to evaluate in a rigorous and thorough way, more than any other published study, whether resection of symptomatic pineal cysts (SPCs) is safe and effective in improving symptoms and quality of life of patients with SPCs. We also hope to understand how your symptoms may be related to the pineal cyst.

The study will be conducted in Cambridge. Patients will be evaluated by a neurosurgeon experienced in treating patients with SPCs to determine whether the symptoms patients suffer are likely to be related to the pineal cyst. If so, patients will be counselled about the likelihood of improvement and risks of surgery and if they wish, they will be offered an operation to resect (surgically remove) their cyst.

If patients wish to proceed, they will be seen by a neurologist, neuro-ophthalmologist and will undergo evaluation by a neuro-psychologist. They will also have a new MRI scan and computerised measurement of intracranial pressure. To do this, patients will have a wire inserted under general anaesthetic and the pressure will be measured by a computer overnight.

Some time later, the surgery to resect the pineal cyst will then be carried out. After surgery, two visits to Cambridge will be required: one at 3 month and one 12 months after surgery. At 3 months, they will only be seen by a neurosurgeon. At 12 months patients will be seen by a neurosurgeon, ophthalmologist (only if they had any visual symptoms prior to surgery) and neuropsychologist.

For a patient to be considered for this study they must:

1. Have a pineal cyst that is larger than 9mm in any dimension
2. Be 18 years or older
3. Have 2 or more or the following (groups of) symptoms:
a) Headaches/pressure in the head-like sensation
b) Visual symptoms, e.g. blurred vision, double vision, light sensitivity
c) Hearing problems
d) Balance problems, e.g. poor balance,
e) Speech/memory problems, e.g. difficulty remembering words and events, word finding difficulties
d) Feeling of disconnection from the surrounding, woolly brain, foggy brain

About our study

* If this study was to go ahead, is this something you would be willing take part in?

Definitely yes	Very likely	Possibly	Unlikely	Definitely no
◯	◯	◯	◯	◯

Please briefly explain your reasons why

* Would you be willing to travel to Cambridge for assessment and, if indicated, for surgery?

- ◯ Yes
- ◯ No

If no, please state reason(s) below.

Do you have any comments about the design of the proposed study? For example, what you like and dislike, what you would like to change etc.

* What do you think of the current state of management of symptomatic pineal cysts in the UK?

- ◯ Excellent there is nothing to improve
- ◯ Good but some improvement is required
- ◯ Unsatisfactory and major improvement is required
- ◯ Very unsatisfactory major overhaul is required
- ◯ I am not sure
- ◯ Other (please specify)

Please write any observations, comments, suggestions management of symptomatic pineal cysts in the UK.

Thank you

Thank you for participating in this questionnaire. We truly appreciate your time.
