# Supplementary material for: International web-based survey of patients with non-hydrocephalic symptomatic pineal cysts
Source: Acta Neurochir (Wien). 2024 Dec 28;166(1):509. doi: 10.1007/s00701-024-06403-5 (PMC11682015; doi:10.1007/s00701-024-06403-5)

**Supplementary figures/tables**

Supplemental Table 1: Types of symptoms measured

| **Label** | **Symptom as described in survey** |
| --- | --- |
| Headache | Headache or “pressure-in-the-head”-like sensation |
| Migraine | Migraine-like headaches |
| Balance | Balance problems |
| Disconnect | Feeling of disconnection |
| Faint | Any episode of fainting |
| Speech | Speech problems |
| Seizure | Any seizure/epileptic attacks |
| Sleep | Sleep problems |
| Hearing | Hearing problems |
| Vision | Visual symptoms |
| Memory | Cognitive/memory problems |
| Fatigue | Any low energy levels/fatigue |
| Sensation | Sensation problems |

Supplemental Table 2: Results of the linear regression analysis for predictors of cyst size


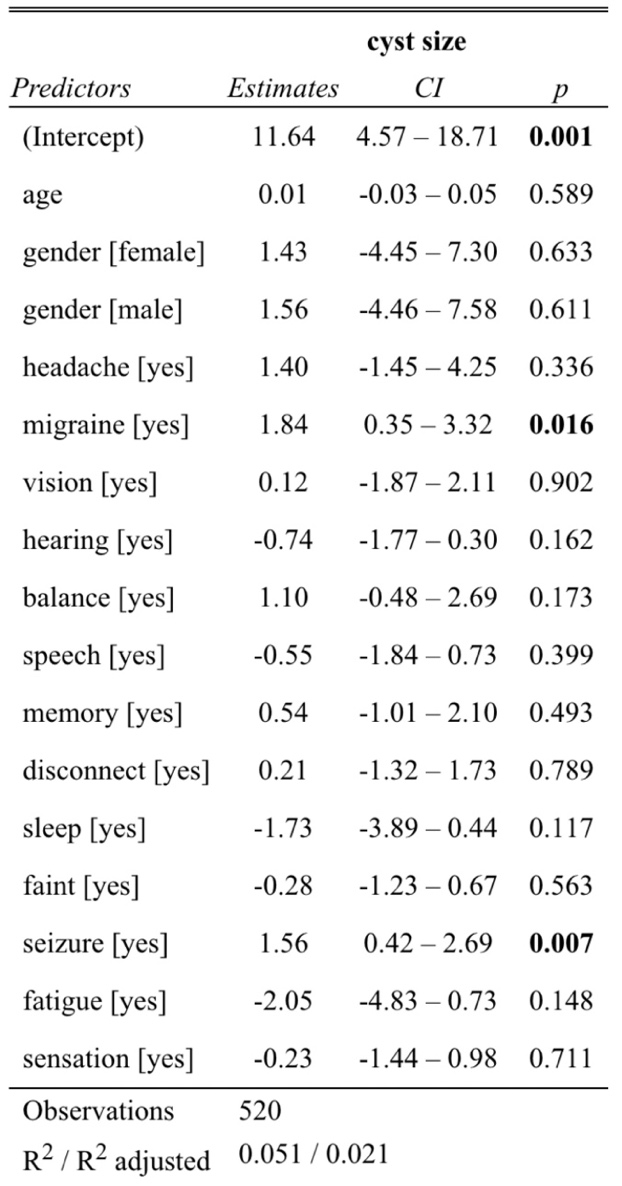

Supplement: Supplementary file 2 — Supplementary file2 (DOCX 180 KB) [file 701_2024_6403_MOESM2_ESM.docx]
